# Induction of Apoptosis in Human Promyelocytic Leukemia HL60 Cells by Panaxynol and Panaxydol

**DOI:** 10.3390/molecules16075561

**Published:** 2011-06-29

**Authors:** Zhonghong Yan, Ruolin Yang, Yi Jiang, Zhihui Yang, Junrui Yang, Qian Zhao, Yang Lu

**Affiliations:** 1 Department of Pharmacy, Shanghai Jiao Tong University School of Medicine, Shanghai 200025, China; 2 Department of Pathophysiology, Shanghai Jiao Tong University School of Medicine, Shanghai 200025, China; 3 Center of Neuroscience Excellence, Louisiana State University Health Sciences Center, New Orleans, LA 70112, USA

**Keywords:** panaxynol, panaxydol, apoptosis, promyelocytic leukemia cell

## Abstract

Panaxynol and panaxydol are naturally occurring polyacetylenes, isolated from the lipophilic fractions of *Panax notoginseng*, that exert anti-proliferative effects against malignant cells. However, to the best of our knowledge, no study concerning the inhibitory effects of the two polyacetylenes on cell growth of human promyelocytic leukemia cells has been reported. In this paper, we examined the antiproliferation and proapoptotic effects of panaxynol and panaxydol on HL60 cells and investigated their mechanism of action. Cell growth inhibition of panaxynol and panaxydol were determined by trypan blue dye exclusion assays. Apoptosis of cells was revealed by morphological observation, analysis for nuclear DNA distribution and by annexin V-FITC/ PI staining using flow cytometry. It was found that panaxynol and panaxydol markedly inhibited proliferation of HL60 cells in a time- and dose-dependent manner via an apoptotic pathway. In concern with these ﬁndings, Western blot analysis showed proteolytic activation of PKCδ, caspase-3 activation and cleavage of poly (ADP [adenosine diphosphate]-ribose) polymerase in HL60 cells treated by panaxynol and panaxydol. In conclusion, panaxynol and panaxydol have profound effects on growth and apoptosis of HL60 cells, suggesting those substances are worthy of further exploration as potential anti-cancer agents.

## 1. Introduction

Human leukemia is one of the common hematological malignant diseases and a major leading cause of human death. The current primary treatment for leukemia is anticancer drug-based chemotherapy that uses one or more drugs to destroy cancer cells, often accompanied by the development of drug resistance and severe side effects [1,2]. Therefore, it is imperative to develop other potential therapeutic agents for the treatment of this disease.

**Figure 1:**
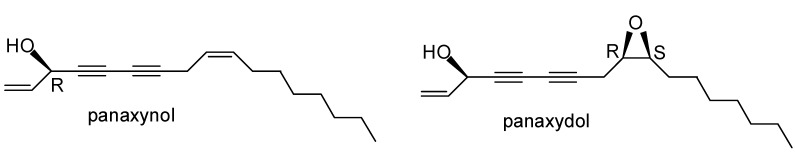
Chemical structures of panaxynol and panaxydol.

Polyacetylenes constitute a distinct group of naturally occurring products, whose numerous pharmacological properties have been recognized [3,4]. Panaxynol (PNN, falcarinol, [3(*R*)-(9*Z*)-heptadeca-1,9-dien-4,6-diyn-3-ol]), and panaxydol (PND, [(3*R*)-9,10-epoxy-1-ene-4,6-diyn-3-ol]), (Figure 1), two of the most abundant bioactive members among this family, have been identified not only in traditional Asian herbal medicines, e.g., *Panax ginseng*, *P. quinquefolium*, *P. notoginseng* and *P. japonicus*, but also in common dietary plants, e.g., carrots, celery, fennel, parsley and parsnip [5]. The effects of these polyacetylenes towards human cancer cells, their human bioavailability and ability to reduce tumour formation in a mammalian *in vivo* model indicated that they may also provide health benefits [6]. Previous studies have shown that PNN or PND possess antiproliferation effects on various cancer cell lines including colon cancer, renal cell carcinoma, malignant melanoma, ovarian carcinoma, and hepatocellular carcinoma [7,8,9]. In addition, PND also induces apoptosis preferentially in cancer cells including a human leukemia T cell line Jurkat, a human breast cancer cell line MCF-7, 1170-I (tumorigenic) cell line and rat glioma line C6, suggesting its potential as a cancer therapeutic agent. [10,11]. However, sensitivity of human promyelocytic leukemia cells to PNN and PND has not been reported. Preliminary work in our laboratory showed the differentiation induction of PNN [12], and here the apoptosis induced by PNN and PND were investigated.

In the present work, PNN and PND were initially isolated from lipophilic fractions of *Panax notoginseng*, a well-known Chinese traditional medicine used for promoting blood circulation. We studied the antiproliferation effect of PNN and PND on human promyelocytic leukemia HL60 cells, and found that PNN and PND inhibited the growth of HL60 cells signiﬁcantly in a time- and dose-dependent manner via apoptotic pathway, involved proteolytic activation of PKCδ, caspase-3 activation and cleavage of PARP.

## 2. Results and Discussion

### 2.1. Effects of PNN and PND on Cell Growth

**Figure 2:**
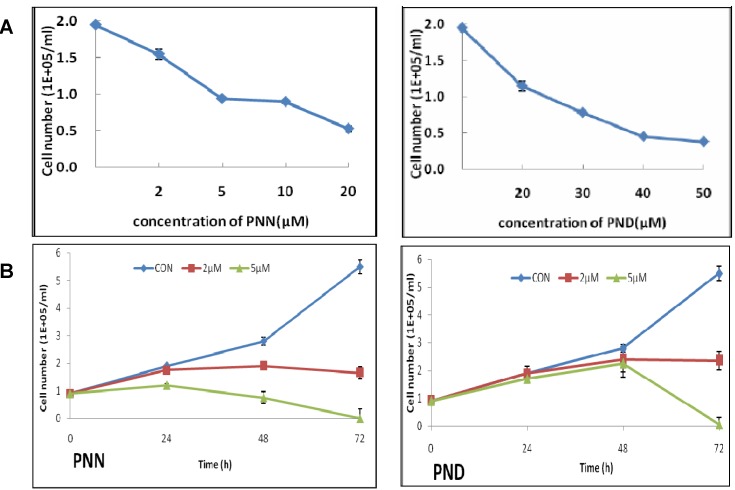
Effects of PNN and PND on the growth of HL60 cells. HL60 cells were incubated with the indicated concentrations of PNN or PND. The growth curve of HL60 cells after treatment with PNN (left) and PND (right) for 24 h (**A**) and 72 h (**B**). Every point represents the mean of triplicate samples.

The effects of PNN and PND on HL60 cell growth inhibition were determined by trypan blue dye exclusion assay. As shown in Figure 2A, HL60 cells were treated with different concentrations of PNN and PND for 24 h. The result showed that, PNN and PND strongly inhibited cell growth in a dose-dependent manner. To test whether long-term treatment would increase inhibition efficiency, after treatment for 24–72 h with low concentration (2 or 5 μM) of PNN and PND respectively, cell growth inhibition was time related. Short-term treatment (<48 h) resulted in a slight cell growth inhibition and a steep rise in the percentage inhibition was observed between 48 and 72 h. Especially at 72 h after treatment with 5 μM PNN or 5 μM PND, almost all cells died. According to Figure 2A, 5 μM PNN and 30 μM PND resulted in 51.8% ± 1.8% and 58.3% ± 1.6% cell growth inhibition after 24 h of treatment. Therefore, the cells treated with 5 μM PNN and 30 μM PND were adopted for subsequent experiments.

### 2.2. Morphological Analysis

**Figure 3:**
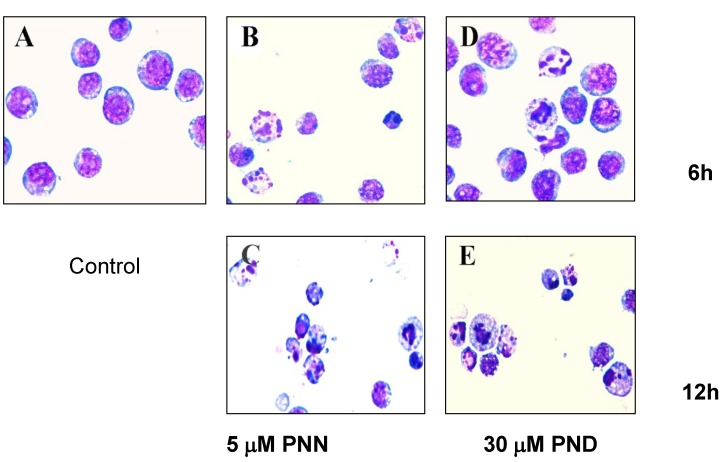
Representative fields showing apoptotic cells. HL60 cells were treated without (**A**) and with 5 μM PNN for 6 h (**B**) and 12 h (**C**), with 30 μM PND for 6 h (**D**) and 12 h (**E**). Cells were centrifuged onto slides by cytospin (Shandon, Runcorn, 500 rpm, 4 minutes), stained with Wright’s staining, and examined under an Olympus BX60 microscope (Olympus, Tokyo, Japan).

To investigate whether the inhibition of cell growth by PNN and PND was related to apoptotic cell death, morphological observation of HL60 cells was performed, and the results showed that the targeted cells underwent gross morphological changes. After treatment with 5 μM PNN or 30 μM PND for 6 and 12 h, the cells showed typical apoptotic changes including chromatin condensation, nuclear fragmentation and formation of apoptotic bodies (Figure 3), suggesting that PNN and PND induced cell death by way of apoptosis. Also, the level of apoptosis gradually increased in a time-dependent manner.

### 2.3. Apoptosis Assays Using Flow Cytometer

**Figure 4:**
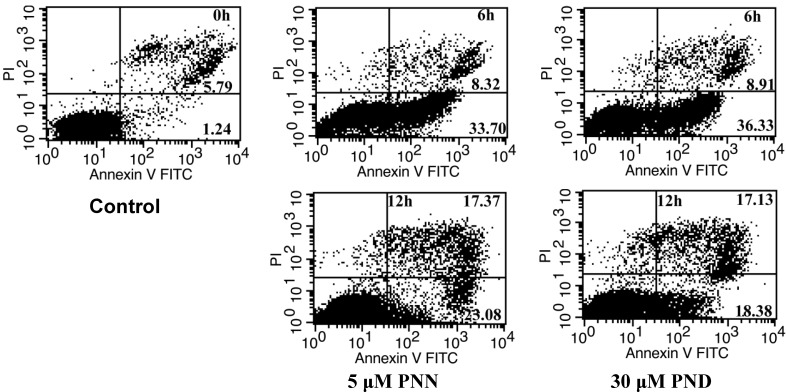
Detection of cell apoptosis using flow cytometry after annexin V-FITC/PI staining. Lower left quadrant, viable cells (annexin V−/PI−); lower right quadrant, early apoptotic cells (annexin V+/PI−); upper right quadrant, late apoptotic cells (annexin V+/PI+); upper left quadrant, necrotic cells (annexin V−/PI+). Cells were exposed to 5μM PNNor 30μM PND for 0, 6 and 12 h. The numbers in panel represent the percentage of annexin-V+/PI− and annexin-V+/PI+ cells. Data were representative of three independent experiments.

To confirm that PNN and PND induce apoptosis, annexin V-ﬂuorescein isothiocyanate (FITC) and propidium iodide (PI) double staining-based ﬂow cytometry analysis, the most sensitive and the most speciﬁc test for determining apoptotic cells in suspension culture [11], was performed on HL60 cells. PI was used to differentiate apoptotic cells with membrane integrity (annexin V+/PI−) from that had lost membrane integrity (annexin V+/PI+). After administration of PNN and PND at different concentrations for 24 h, most of apoptotic cells were in the upper right quadrant (annexin V+/PI+) indicating that nearly all apoptotic cells were in the late stage of apoptosis (data not shown). To detect early apoptotic cells, the treating time were shortened. The result showed that, after 5 μM PNN and 30 μM PND treatment for 6 h, the early apoptotic cells in the lower right quadrant (annexin V+/PI−) were observed (Figure 4). At assay time of 12 h, the population of cells indicated a shift from viable cells to early stage and late stage apoptosis, and the percentage of apoptotic HL60 cells were increased in a time-dependent manner. These results were in agreement with morphological data.

### 2.4.Cell Cycle Analysis

**Figure 5:**
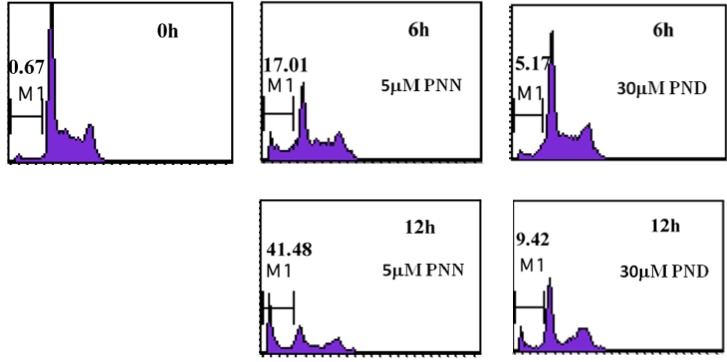
Flow cytometry analyses of nuclear DNA content. HL60 cells were treated with 5μM PNN and 30 μM PND for the indicated time, the numbers in panel represent the M1 (sub-G1, hypodiploid) region, *i.e.*, the percentage of apoptotic cells and the values were the mean of triplicate.

It is widely accepted that apoptotic cells have reduced DNA stability due to degradation and subsequent leakage from cells [14]. These DNA can be stained by propidium iodide (PI). Thus, the appearance of cells with low stability (*i.e.*, hypoploid cells or sub-G1 cells) in cultures has been considered to be another marker of apoptosis and has been used to quantify the extent of apoptosis. To show in more detail that the inhibition of cell growth by PNN and PND is closely related to apoptosis, nuclear DNA distribution was analyzed on ﬂow cytometry. The treatment of HL60 cells with 5 μM PNN and 30 μM PND induced an increase in the percentage of hypoploid cells from 17.01% to 41.48% and 5.17% to 9.42% within 6-12 h, respectively, compared with the untreated cells that showed 0.67% (Figure 5). There were significant differences between the treated group and the control group after the same duration (P < 0.05). It was shown that PNN and PND induced a time-dependent increase in hypoploid cells, an important indication of cells becoming apoptotic. Taken together, we concluded that PNN and PND inhibited HL60 cells growth through induction of apoptosis.

### 2.5. Apoptosis-Related Proteins in HL60 Treated by PNN and PND

**Figure 6:**
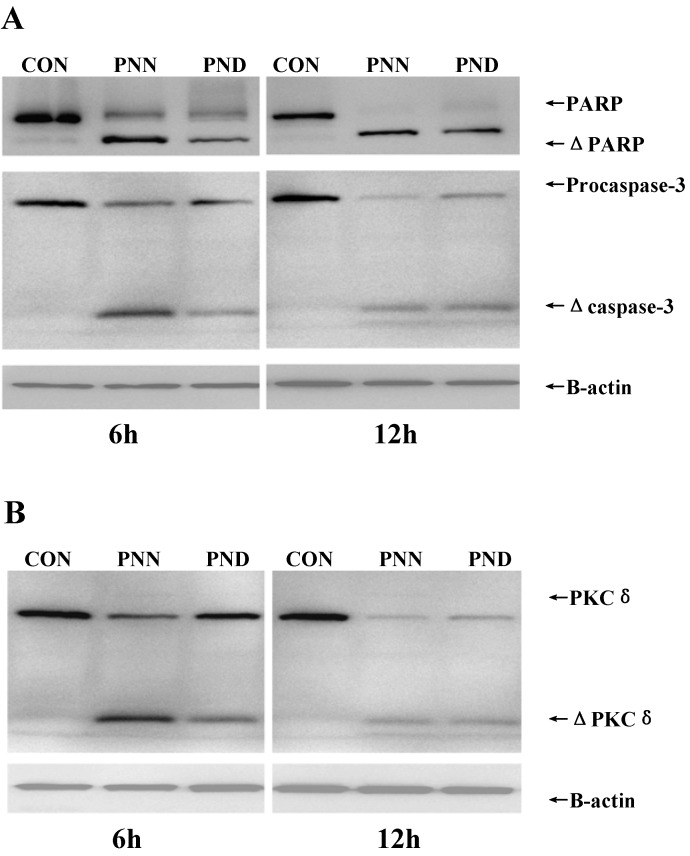
Expression levels of apoptosis-related proteins after the treatment of 5μM PNN and 30μM PND for 6h and 12h in HL60 cells. Proteins were analyzed by SDS-PAGE and western blot using caspase-3, PARP (**A**) and PKCδ (**B**) antibodies. β-actin was used as a control for loading. The presented blot was representative of three independent experiments.

To further elucidate the mechanism(s) of PNN- or PND-induced apoptotic cell death, expression of apoptosis-related proteins in HL60 cells was measured. Caspase-3 activation and PKCδ cleavage were detected after 6, 12 h of incubation with 5 μM PNN or 30 μM PND, but not observed at 1, 2, 4 h (data not shown). Activation of the caspase cascade, e.g., caspase-3, appears to be a crucial event during the apoptotic process. Thus, we examined the activation of caspase-3 by Western blot analysis with specific antibodies. Intact caspase-3 was a 32 kDa protein as detected in control cells and was processed into its catalytically active p17 subunits in treated HL60 cells (Figure 6A). To confirm the activation of caspase-3, we evaluated the cleavage of Poly (ADP-ribose) polymerase (PARP), a major substrate of caspase-3. PNN or PND treatment caused the cleavage of 116 kDa PARP into 85 kDa form (Figure 6A), indicating that caspase-3 was indeed activated during PNN- or PND-induced HL60 cell apoptosis. Caspase-3 is an effector caspase whose activation is critical for the execution of apoptotic death of tumor cells [15,16]. It was shown clearly that caspase-3 was activated during PNN- or PND- induced HL60 cell apoptosis and the activation was further verified by the cleavage of PARP, a preferred substrate of caspase-3 in apoptotic cells, suggesting that the activation of caspase-3 was a common mechanism responsible for PNN- or PND-induced apoptosis.

Protein kinase C δ (PKCδ), a ubiquitously expressed member of the novel PKC family, is activated by translocation, tyrosine phosphorylation, or proteolytic cleavage into 41 kDa catalytically active fragment, and has been implicated in many important cellular processes, including regulation of apoptotic cell death as a pro-apoptotic isoform [17]. Studies using PKCδ knockout mice for γ-irradiation-induced apoptosis demonstrated that PKCδ is required for execution of the apoptotic programme [18]. In addition, increasing literatures supported a direct link between cleavage of PKCδ and activation of caspase-3 [19,20,21,22,23,24], of which the relationship remains unclear. Some reports indicated that caspase-3 cleaved PKCδ [19,20,21], whereas certain studies showed PKCδ acted upstream of caspase-3, and pointed to the existence of a positive regulatory loop between PKCδ and caspase-3 [19,22,23,24]. This variance might depend on the nature of stimuli and cell-type speciﬁc. In the present study PKCδ expression was detected by Western blot. Figure 6B showed that PNN and PND induced a rapid proteolytic cleavage of PKCδ into a 41 kDa fragment. These results indicated that the apoptosis proceed induced by PNN and PND might be mediated through the proteolytic activation of PKCδ, implicated primarily in the apoptotic cell death of many cellular systems. However whether PKCδ is the upstream or the downstream of the caspase-3 need further studies.

In this research, we found that PNN and PND possess similar inhibition of proliferation of HL60 cells by a common mechanism. The difference of the growth inhibition dose implied that HL60 cells was more sensitive to PNN, which might result from the difference of structures. To define reliable apoptosis inducing effect of PNN and PND, the related work concerning more cell lines is currently underway.

## 3. Experimental

### 3.1. General

RPMI-1640 medium, fetal bovine serum (FBS), trypan Blue, dimethylsulfoxide (DMSO), and propidium iodide (PI) were purchased from Sigma Chemical (St. Louis, MO, USA). ApoAlert Annexin-V kit and all antibodies were purchased from BD Biosciences (San Diego, CA, USA). All spectral data were obtained on the following instruments: ^1^H- and ^13^C-NMR on a Bruker AM-400 in CDCl_3_ with TMS as internal standard, and MS on a Waters Q-701 micro mass spectrometer. Sanqi (*P. notoginseng* (Burk.) F. H. Chen) was purchased from Xu Chongdao Traditional Chinese Medicine Co. Ltd.

### 3.2. Extraction and Isolation

Air-dried roots of *Panax notoginseng* (1 kg) were powered and extracted with 95% EtOH (2 L × 3) three times. After removal of solvent, the residue (158.8 g) was partitioned between petroleum ether and water to give a petroleum ether extract (7.1 g), which was subjected to silica gel column chromatography, eluting with petroleum ether (60–90 °C) containing increasing concentrations of ethyl acetate (EtOAc). Each fraction was 50 mL. On the basis of the thin-layer chromatography (TLC) profiles, fractions 46–52 (petroleum ether/EtOAc 20:1) and 54–58 (petroleum ether/EtOAc 20:1) were then subjected to further silica gel column chromatography and preparative TLC, respectively, which yielded PNN and PND.

### 3.3. PNN and PND Standards

Authentic standards of the polyacetylenes PNN [3(*R*)-(9*Z*)-heptadeca-1,9-dien-4,6-diyn-3-ol] and PND [(3*R*)-9,10-epoxy-1-ene-4,6-diyn-3-ol] were isolated from 1 kg of *P. notoginseng* roots according to the procedure described by our laboratory [25]. They were identified by UV, mass spectrometry (MS), and NMR (^1^H- and ^13^C-), and the full spectral data sets were in accordance with the published values [26,27,28,29]. The purity checked by HPLC was above 98%.

*Panaxynol.* A colorless oil, ESI-MS: m/z 267 [M+Na]^+^; ^1^H-NMR (δ): 5.94 (1H, *ddd*, *J* = 16.3, 10.2, 5.4 Hz; H-2), 5.52 (1H, *dd*, *J* = 9.1, 7.7 Hz; H-10), 5.46 (1H, *d*, *J* = 17.1 Hz; H-1a), 5.37 (1H, *dd*, *J* = 12.3, 7.1 Hz; H-9), 5.24 (1H, *d*, *J* = 10.1 Hz; H-1b), 4.91 (1H, *d*, *J* = 5.1 Hz; H-3), 3.03 (2H, *d*, *J* = 6.9 Hz; H-8), 2.02 (2H, *dd*, *J* = 14.4, 7.2 Hz; H-11), 1.36 (2H, *t*, *J* = 6.6 Hz), 0.88 (3H, *t*, *J* = 6.8 Hz; H-17); ^13^C-NMR (δ): 136.2 (C-2), 133.1 (C-10), 121.9 (C-9), 117.0 (C-1), 80.3 (C-7), 74.3 (C-4), 71.3 (C-5), 64.0 (C-6), 63.5 (C-3), 31.8 (C-15), 29.2 (C-12), 29.2 (C-13), 29.1 (C-14), 27.2 (C-11), 22.6 (C-16), 17.9 (C-8), 14.1 (C-17).

*Panaxydol.* A yellow oil, ESI-MS, m/z 283 [M+Na]^+^; ^1^H-NMR (δ): 5.95 (1H, *ddd*, *J* = 17.1, 10.2, 5.4 Hz, H-2), 5.48 (1H, *d*, *J* = 17.1 Hz, H-1a), 5.26 (1H, *d*, *J* = 10.2 Hz, H-1b), 4.93 (1H, *brs*, H-3), 3.15(1H, *ddd*, *J* = 4.1, 5.5, 7.1 Hz, H-9), 2.97(1H, *ddd*, *J* = 4.2, 5.4, 6.1 Hz, H-10), 2.71(1H, *dd*, *J* = 5.5, 17.8 Hz, Hb-8), 2.39 (1H, *dd*, *J* = 7.1, 17.8 Hz; Ha-8), 1.4-1.6 (4H, *m*, H-11, 12), 1.25–1.37 (8H, *m* ), 0.89 (3H, *t*, *J* = 6.9 Hz; Me-17); ^13^C-NMR (δ): 136.1 (C-2), 117.3 (C-1), 76.7 (C-7), 74.9 (C-4), 71.0 (C-5), 66.3 (C-6), 63.6 (C-3), 57.1 (C-10), 54.4 (C-9), 31.8 (C-15), 29.5 (C-13), 29.3 (C-14), 27.6 (C-11), 26.5 (C-12), 22.7 (C-16), 19.5(C-8), 14.2 (C-17).

### 3.4. Cell Culture and Drug Treatments

Human acute promyelocytic leukemia cell line HL60 was obtained from Cell Bank of Shanghai Institutes for Biological Sciences (Shanghai, China). The cells were cultured in RPMI-1640 medium (Sigma, St Louis, MO) supplemented with 10% heat-inactivated fetal calf serum (FCS; HyClone, Logan, UT) in a 5% CO_2_ 95% air humidified atmosphere at 37 °C. For experiments, a starting inoculum 1 × 10^5^ HL60 cells/ml in the exponential phase of growth was used and treated with the appropriate drugs at the indicated concentrations. PNN and PND were dissolved in 100% DMSO, stored in dark at –20 °C and brought to room temperature before use. Final concentration of DMSO never exceeded 0.5% (v/v) in either control or treated cells. Comparison of the control cells treated with 0.5% DMSO and control cells without DMSO treatment did not show any difference in all experiments.

### 3.5. Cell Viability Assay

The viability of cells was determined by trypan blue dye exclusion assay. Cells were seeded at density of 1 × 10^5^ cells/well in triplicates onto 24-well plates, then, PNN and PND were added to medium at indicated concentration respectively. At assay time, cells were collected, mixed with an equal volume of PBS containing 0.4% trypan blue dye, and counted in a hemocytometer. Actual cell numbers were calculated by multiplying diluted times compared with initial cell numbers. Cell viability% = viable cell numbers/total (viable + dead) cell numbers ×100%. Inhibition% = (control groups - experimental groups)/control groups of viable cell numbers×100%.

### 3.6. Morphological Analysis

After being treated with 5 μM PNN and 30 μM PND for 0, 6, 12 h, the HL60 cells (4 × 10^4^) were washed with PBS for three times then collected onto slides by cytospin (Shandon, Runcorn, UK), stained with Wright’s staining, and examined under light microscope (Olympus, BX60, Japan).

### 3.7. Apoptosis Assays Using Flow Cytometer

Annexin-V assay was performed on a ﬂow cytometry (Beckman Coulter) according to the instructions provided with the ApoAlertAnnexin-V kit (BD Biosciences, USA). Briefly, HL60 cells (1 × 10^6^) was treated with 0.1% DMSO, 5 μM PNN and 30 μM PND for indicated times at 37 °C. The treated cells were harvested by centrifugation, washed with PBS, resuspended in binding buffer (100 mM HEPES pH 7.4, 1.5 mM NaCl, 50 mM KCl, 10 mM MgCl_2_, 18 mM CaCl_2_), and incubated with 5 μL of annexin-V and 10 μL of 50 μg/mL PI for 15 min in the dark. Cells ﬂuorescence was measured on FACScan ﬂow cytometer using an argon ion laser (488 nm) and examined on the CellQuest Pro IVD software.

### 3.8. Nuclear DNA Content Distribution

To assess the distribution of nuclear DNA content, HL60 cells (1 × 10^6^) were collected, rinsed, and ﬁxed overnight in 70% cold ethanol at 4 °C. Then, fixed cells were resuspended in 1 mL PBS (pH 7.4), treated with RNase (50 μg/mL) for 30 min at 37 °C, and stained with 50 μg/mL PI (Sigma) for 15 min at 4 °C in dark. Cell-cycle distribution was determined by ﬂow cytometry (Beckman Coulter, Miami, FL). All data were collected, stored, and analyzed by Multicycle software (Beckman Coulter).

### 3.9. Western Blotting Analysis

Cells were washed with PBS and lysed with lysis buffer (0.01 M Tris-HCl (pH 7.2), 0.1 M DTT (dithiothreitol), 2% SDS (sodium dodecyl sulfate), 10% glycerol). Cell lysates were centrifuged at 12,000 rpm for 5 minutes at 4 °C, and proteins in the supernatants were quantiﬁed according to the Bradford method. Protein extracts were equally loaded to 12% SDS-polyacrylamide gel, electrophoresed, and transferred to nitrocellulose membrane (Amersham Bioscience, Buckinghamshire, UK) by electroblotting. The blots were stained with 0.2% Ponceau S red to ensure equal protein loading. After blocking with 5% nonfat milk in PBS, the membranes were probed with anticleaved caspase-3 (1:1000; Cell Signaling), PARP (1:500, F2, Santa Cruz Biotech), and anti-PKCδ (1:2000, C-20; Santa Cruz Biotech), followed by horseradish peroxidase (HRP)-linked secondary antibodies (Cell Signaling). The signals were detected by chemiluminescence phototope-HRP kit (Cell Signaling) according to manufacturer’s instructions. As necessary, blots were stripped and reprobed with β-actin as an internal control.

### 3.10. Statistical Analysis

The data were expressed as the means ± SD. The signiﬁcance of the difference between groups was determined by the Student’s t-test. A probability value of P < 0.05 was considered to be statistically significant.

## 4. Conclusions

In conclusion, PNN and PND exhibited antiproliferative effects in a time- and dose-dependent manner on human promyeloid leukemia HL60 cells by induction of apoptosis that was associated with proteolytic cleavage of PKCδ, caspase-3 activation and degradation of PARP. Apoptosis is one of main therapeutic targets in cancer research, so it appears reasonable to suggest that PNN and PND may have potential as agents with chemotherapeutic and cytostatic activity against human leukemia. However, further investigation of their activity, *in vivo*, is necessary to elaborate and exploit this promise.
